# 

**Published:** 1894-05

**Authors:** 


Vol. XLVIII. MAY, 1894. No. 5.

### THE

### DENTAL REGISTER

A MONTHLY JOURNAL.

DEVOTED TO THE INTERESTS OF THE PROFESSION.

EDITED BY

J. TAFT. D.D.S.

ASSOCIATE EDITORS,

WILL. H. WHITSLAR, D.D.S. N. S. HOFF, D.D.S.

PUBLISHED BY

SAM'L A. CROCKER & CO.,

SUCCESSORS TO SPENCER & CROCKER,

OHIO DENTAL AND SURGICAL DEPOT,

Nos. 117, 119, 121 WEST FIFTH STREET, CINCINNATI, OHIO.

**TERMS:—$2.00 per Annum, in Advance. Single Copies, 25 cts.**

